# Novel Fluorinated Spermine and Small Molecule PEI to Deliver Anti-PD-L1 and Anti-VEGF siRNA for Highly Efficient Tumor Therapy

**DOI:** 10.3390/pharmaceutics13122058

**Published:** 2021-12-02

**Authors:** Yihui Zhang, Zihan Yuan, Yi Jin, Wenkai Zhang, Wei-En Yuan

**Affiliations:** Engineering Research Center of Cell & Therapeutic Antibody, Ministry of Education, School of Pharmacy, Shanghai Jiao Tong University, Shanghai 200240, China; jiaodazhangyihui@sjtu.edu.cn (Y.Z.); yzh-32@sjtu.edu.cn (Z.Y.); jinyi1997@sjtu.edu.cn (Y.J.); zhangwenkai@sjtu.edu.cn (W.Z.)

**Keywords:** fluorine modified carrier, gene silencing, VEGF, PD-L1, anti-tumor

## Abstract

Small interfering RNA (siRNA) can specifically silence disease gene expression. This project investigated the overexpression of programmed death receptor ligand 1 (PD-L1) and vascular endothelial growth factor (VEGF) on the surface of tumor cells. However, the main obstacle to the development of gene therapy drugs is the lack of an efficient delivery vector, which should be able to overcome multiple delivery barriers and protect siRNA to enter the target cells. Therefore, a novel fluorine-modified endogenous molecular carrier TFSPEI was constructed by linking fluorinated groups with hydrophobic and hydrophilic characteristics on the surface of PEI and spermine. The results showed that lower toxicity, higher endocytosis, and silencing efficiency were achieved. We found that the inhibition of VEGF targets can indirectly activate the immune response to promote the tumor-killing and invasion effects of T cells. The combined delivery of anti-VEGF siRNA and anti-PD-L1 siRNA could inhibit the expression of corresponding proteins, restore the anti-tumor function of T cells and inhibit the growth of neovascularization, and obtained significant anti-tumor effects. Therefore, this safe and efficient fluorinated spermine and small molecule PEI-based anti-PD-L1 and anti-VEGF siRNA delivery system is expected to provide a new strategy for gene therapy of tumors.

## 1. Introduction

In recent years, the incidence of tumors has been increasing year by year, seriously threatening human health. The drugs used to treat tumors are still concentrated on traditional small-molecule chemical drugs and macromolecular protein drugs [1], but the targets of these drugs only account for 2–5% of the whole genome [2], and more than 90% of the remaining genome requires gene drugs to achieve target druggability. By the end of 2017, nearly 2600 gene therapy clinical trials were underway or have been approved globally, 65% of which are used for the treatment of cancer [3,4].

In most malignant tumors, continuous angiogenesis is regarded as an important tumor marker [5,6]. Among the factors promoting tumor angiogenesis, VEGF has been proved to be the most important, which can stimulate the growth of vascular endothelial cells and hinder the effective delivery of anti-tumor drugs to tumor tissues [7,8,9]. In recent years, many studies have shown that VEGF can also induce the proliferation and differentiation of regulatory cells and myeloid-derived suppressor cells, inhibit the maturation of dendritic cells (DCs), and induce the expression of immunosuppressive molecules PD-L1 in DCs, thereby inhibiting the body’s immune response to tumor cells [10,11,12,13,14]. In addition, PD-L1 is highly expressed in tumor cells and tumor-associated activating antigen-presenting cells, and programmed cell death protein 1 (PD-1) is highly expressed in tumor-infiltrating lymphocytes under long-term stimulation of tumor antigens [15]. The combination of PD-L1 and PD-1 can induce T cell apoptosis and exhaustion, inhibit the activation, proliferation, and anti-tumor function of tumor antigen-specific CD8-positive T cells and realize the immune escape of tumor cells [16,17,18]. Therefore, the combined inhibition of PD-L1 and VEGF expression can effectively activate the immune response, promote the tumor infiltration effect of T cells, and inhibit tumor angiogenesis, making it an ideal target for combined therapy [10,19].

Small interfering RNA (siRNA) is a short-chain (21–23 bp) RNA that can specifically block gene expression in messenger RNA (mRNA) [20,21]. It can correspond to therapeutic gene targets, and specifically silence genes in the transcriptional and post-transcriptional stages [22,23]. However, the clinical use of siRNA is limited by factors such as being easily degraded by ribonuclease (RNase), and that it is difficult to be taken up by cells [24]. Therefore, a nucleic acid delivery carrier that can break through a series of barriers and ensure safe and efficient delivery is essentiall [25]. Non-virus carriers with high security and ease to be modified have been widely studied [26]. Non-viral vector transfection reagents include anionic liposomes, cationic liposomes, inorganic nanomaterials, and other cationic compounds synthesized for cell transfection [27]. Among them, cationic liposomes and cationic polymers are the main types of cationic transfection reagents for non-viral carriers. Polyethyleneimine (PEI), as a classical non-viral carrier, has great potential for structural modification and has a good ability to compress and transport nucleic acids [28]. This is due to its high amino density and strong buffering capacity in acidic environments. The transfection ability of PEI is highly correlated with charge density, and it is generally believed that the transfection efficiency increases with the increase of molecular weight. Moreover, increasing the nitrogen to phosphorus (N/P) ratio of the “PEI-nucleic acid” polyplex can also enhance cell transfection ability [29]. However, there is usually a positive correlation between transfection efficiency and cytotoxicity [30]. Due to siRNA’s low surface charge density, the polyplex structure formed with delivery carriers is relatively loose, is easily replaced by negative electrolyte in plasma and also reduces the delivery efficiency [31,32].

To solve these problems, it is feasible to modify the PEI structure by hydrophobic groups such as hydrocarbon alkyl chain and cholesterol, which can significantly improve the stability of polyplex in an aqueous solution. However, such polyplexes are unstable in the phospholipid cell membrane and are easily disturbed by the alkyl chain in the phospholipid molecules [33,34,35]. Therefore, a PEI/siRNA polyplex with high efficiency and low toxicity, which can maintain stability in aqueous and hydrophobic phospholipid environments, is the key to the success of gene therapy [36,37]. Recent studies have shown that fluorinated modification can simultaneously improve the stability of polyplex, endocytosis, endosome escape, intracellular nucleic acid release, and antiserum performances, thus greatly increasing the efficiency of gene delivery [38,39,40,41]. This is due to the unique fluorophilic effects of fluorophilic groups, which have high affinity and can produce surface energy, so siRNA can be condensed and delivered at a low mass ratio, achieving high transfection efficiency and low cytotoxicity [42]. Moreover, due to the weak interaction between fluorine-containing groups and phospholipid molecules, it is easier to be taken up by cells, and the disturbance to the cell membrane is correspondingly reduced [43]. As fluoroalkyl is both hydrophobic and hydrophilic, fluorinated polycationic carriers are inert to the proteins and lipids in the serum, showing excellent serum stability in nucleic acid delivery [44,45]. In addition to fluoroalkyl, fluorine-containing aromatic structures also have the same effect, which is directly proportional to the number of fluorine atoms on the aromatic ring in a certain range [40,46].

**Figure 1:**
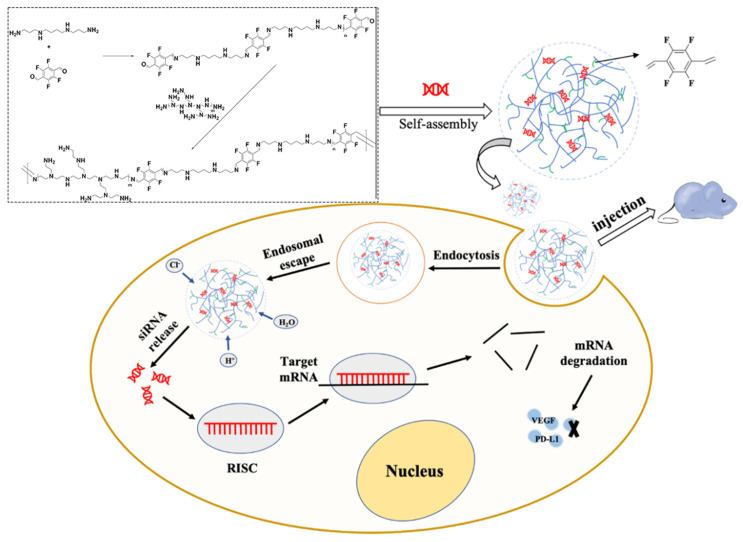
Schematic diagram of experimental principle. Schematic illustration shows the formation of the polyplex and its mode of action in tumor cells.

In this study, 2,3,5,6-tetrafluoroterephthaldehyde was selected to react with PEI 1.8 kDa and spermine, and fluorinated modification was performed by forming imine bonds. The novel fluorinated carrier was used to deliver corresponding siRNA to mediate silencing of the expression of VEGF and PD-L1, effectively restoring the anti-tumor function of T cells and inhibiting tumor angiogenesis through multiple signal pathways. Subsequently, the physicochemical properties of the polyplex were characterized. Anti-PD-1/PD-L1 immune checkpoint blocking therapies have been extensively studied in breast cancer, non-small cell lung cancer, and colorectal cancer [47]. Among them, colorectal cancer has become the third most prevalent tumor and an important factor in the high cancer mortality rate. Therefore, CT26 colorectal cancer cells were taken as the representative cell line to provide a basis for the therapeutic effect of our drug delivery system on tumors. The cytotoxicity and silencing efficiency of the polyplex were investigated in vitro on CT26 cells with high expression of VEGF and PD-L1. Lastly, a tumor-bearing mouse model was established to evaluate the tumor-suppressive effect and toxicity of the gene delivery system (Figure 1).

## 2. Materials and Methods

### 2.1. Materials

Branched polyethyleneimine (PEI; 1.8 and 25 kDa in average Mw), dichloromethane, spermine were purchased from Sigma-Aldrich (St. Louis, MO, USA). 2,3,5,6-Tetrafluoroterephthaldehyde was purchased from Ling Feng (Shanghai, China). Cellulose membranes (MWCO = 3500 Da) were purchased from Thermo Fisher Scientific (Waltham, MA, USA). siRNA was all constructed by GenePharma (Shanghai, China), the sequence of anti-VEGF siRNA was 5′-CUU ACG CUG AGU ACU UCG AdTdT-3′, anti-PD-L1 siRNA was 5′-AGA CGU AAG CAG UGU UGA AdTdT-3′, NC-siRNA was 5′-UUC UCC GAA CGU GUC ACG UdTdT-3′. All the reagents were used as received without further purification.

### 2.2. Methods

#### 2.2.1. Cell Culture

CT26 was ordered from the cell bank of the Chinese Academy of Sciences (Shanghai, China). CT26 was cultured in RPMI-1640 (GIBCO, Grand Island, NY, USA) with 10% fetal bovine serum (FBS; Biological Industries, Kibbutz Beit Haemek, Israel) and 1% penicillin/streptomycin at 37 °C in the cell incubator with 5% CO_2_.

#### 2.2.2. Synthesis and Characterization of TFSPEI

First, PEI 1.8 kDa (180 mg, 0.1 mmoL), spermine (40.5 mg, 0.2 mmoL), and 2,3,5,6-tetrafluoroterephthaldehyde (82.4 mg, 0.4 mmoL) were dissolved in dichloromethane, respectively. 2,3,5,6-Terafluoroterephtaldehyde solution was slowly added to the spermine solution in an ice bath under the protection of nitrogen. After two hours of reaction at room temperature, PEI 1.8 kDa solution was slowly added to the solution. The reaction solution was stirred for 24 h before vacuum filtration and rotary evaporation to remove dichloromethane. The products were then dissolved in deionized water and dialyzed through cellulose membranes (3500 Da MWCO) for 48 h. TFSPEI was obtained after freeze-drying for 48 h and stored at −20 °C.

The structure of TFSPEI was characterized by Fourier transform infrared spectroscopy (FTIR, Bruker Optics FT-IR spectrometer, Munich, Germany.) and proton nuclear magnetic resonance imagery (19F NMR, Baita Xinbao Instrument Factory SHZ-82A, Changzhou, China), respectively. TFSPEI and dried potassium bromide were mixed and ground in an agate mortar and pressed into transparent slices. Dried potassium bromide was used for blank calibration. The blank potassium bromide slice was evenly smeared with a small amount of PEI 1.8 kDa. About 20 mg of TFSPEI and 2,3,5,6-tetrafluoroterephthaldehyde were placed in a centrifugal tube, and deuterium chloroform was used as the solvent. The average molecular weight was confirmed by size exclusion chromatography (SEC, EcoSEC HLC-8321GPC/HT, Japan). TFSPEI and PEI standards (Mw 800, 1300, 1800, 2000, 10,000, 25,000, and 70,000 kDa) were weighed and dissolved in ultra-pure water to form solutions with a concentration of 2.0 mg/mL. Test parameters were set as follows: Column: PL Aquagel–OH 30 8 μm; Mobile phase: water; Mobile phase flow rate: 1 mL/min; Column temperature: 40 °C.

#### 2.2.3. Preparation of TFSPEI Polyplex

TFSPEI and siRNA were dissolved in RNase-free water and diluted into 2 mg/mL and 20 μg/mL as stock solutions, respectively. To obtain various weight/weight (*w*/*w*) ratios, the polyplex was prepared by mixing different volumes of TFSPEI stock solution with siRNA stock solution, followed by incubating at room temperature for 30 min to form a self-assembled polyplex. Similarly, positive control PEI 25 kDa/siRNA polyplex was obtained in the same way.

#### 2.2.4. Agarose Gel Electrophoresis

The ability of TFSPEI to condense siRNA at different *w*/*w* ratios (0.05, 0.1, 0.5, 1, 3, 5, 10) was determined by agarose gel electrophoresis (AGE). PEI 25 kDa/siRNA polyplex (*w*/*w* ratio = 2) was served as the positive control and naked siRNA (40 ng/μL) was served as the negative control. All the samples were carried out on the 3.0% (*w*/*v*) agarose gel in 1 × TAE running buffer and electrophoresed at 120 V and 400 A for 45 min. The bands of siRNA were imaged under UV illumination (Tanon 2500 Gel Image System, Shanghai, China).

AGE was also used to measure the stability of polyplex in 10% FBS. The TFSPEI and siRNA were prepared at the *w*/*w* ratio of 50 and incubated in a 37 °C, 100 rpm incubator. 100 μL samples were collected at 0 h, 3 h, 6 h, 12 h, 24 h, 36 h, and 48 h respectively. Naked siRNA (40 ng/μL) and pure FBS were served as the control.

#### 2.2.5. Particle size, Zeta Potential, and Morphology Measurements

The particle size, polydispersity index (PDI), and zeta potential of polyplex at various *w*/*w* ratios (1, 5, 10, 20, 30, 40, 50, 60) were determined by dynamic light scattering (DLS) and electrophoretic light scattering (ELS, Brookhaven Particle Size Analyzer, Holtsville, NY, USA), respectively. All measurements were repeated three times. The morphology of the polyplex (*w*/*w* ratio = 50) was detected by a 120 kV Transmission Electron Microscope (TEM; Talos L120C G2, Thermo Fisher Scientific, Waltham, MA, USA).

#### 2.2.6. In Vitro Cytotoxicity

Cytotoxicity of TFSPEI polyplex against CT26 was determined by Cell Counting Kit-8 (CCK-8; DOJINDO LABORATORISE, Shanghai, China) assay. CT26 with the complete medium were seeded into 96-well plates (1 × 10^4^ cells/well) and incubated overnight. The medium in each well was replaced by a 50 μL basic medium, and 10 μL TFSPEI polyplex at different *w*/*w* ratios (1, 5, 10, 20, 30, 40, 50, 60) were incubated with CT26 for 4 h and 24 h, respectively. Then, 10 μL CCK-8 and 50 μL fresh basic medium were added for additional incubation. After about 2 h, 96-well plates were measured with a multifunctional microplate reader (SpectraMax M3 Multi-Mode Microplate Reader, Sunnyvale, CA, USA) under the absorbance of 450 nm and reference at 630 nm. To investigate whether the cytotoxicity was caused by TFSPEI alone or was related to the types of compressed siRNA, the three polyplexes used in the subsequent experiments were tested. To keep the N/P ratio of each polyplex constant, three groups were set as follows: TFSPEI-anti-VEGF polyplex group (siRNA = 50% anti-VEGF siRNA + 50% NC-siRNA), TFSPEI-anti-PD-L1 polyplex group (siRNA = 50% anti-PD-L1 siRNA + 50% NC-siRNA) and TFSPEI-combination polyplex group (siRNA = 50% anti-PD-L1 siRNA + 50% anti-VEGF siRNA). PEI 25 kDa-combination polyplex at the same *w*/*w* ratios was prepared as the positive control, and the blank control group which cells treated with PBS were considered as 100% cell viability. Each ratio was repeated 6 times.

#### 2.2.7. In Vitro Endocytosis Efficiency

CT26 with the complete medium were seeded into 24-well plates (6 × 10^4^ cells/well) and incubated overnight. The medium in each well was replaced by a 500 μL fresh medium. Then, 100 μL TFSPEI polyplex (siRNA was labeled with fluorescence Cy5) at different *w*/*w* ratios (10, 20, 30, 40, 50, 60) were incubated with CT26. After 4 h, the cells were collected and positive cell rates were detected by flow cytometry (FCM) (BD LSRFortessa, Franklin Lakes, NJ, USA) to analyze the endocytosis efficiency of cells in the different cultural environments. PEI 25 kDa polyplex at the same *w*/*w* ratios was prepared as the positive control, naked siRNA as the negative control, and PBS as the blank control. Each ratio was repeated 6 times.

#### 2.2.8. Intracellular Uptake

In order to qualitatively analyze the cellular uptake of TFSPEI polyplex, a Confocal Laser Scanning Microscope (CLSM; TCS SP8 STED 3X, Lecia, Wetzlar, Germany) was used to investigate the localization of polyplex (siRNA was labeled with fluorescence Cy5) in CT26. First, CT26 with the complete medium were seeded into 12-well plates (1 × 10^5^ cells/well) containing cell slides (Fisherbrand, MA, USA) and incubated overnight. The medium was then replaced with a 1 mL basic medium and 200 μL polyplex with a *w*/*w* ratio of 50. After incubation for 2 h and 4 h, the cells were washed and added with 2 mL LysoTracker Green (100 nM; Beyotime, Shanghai, China) to label lysosomes for 1 h at 37 °C. Cells were washed again and 1 mL 0.4% trypan blue solution (Thermo Fisher Scientific, Waltham, MA, USA) was used to mark living cells for 2 min. The washed cells were then fixed with 1 mL 4% paraformaldehyde for 30 min. Finally, 2 mL DAPI (2.5 μg/mL; Roche Diagnostics, Mannheim, Germany) was added to label the nuclei at 37 °C for 3 min, and CLSM was used for the study.

#### 2.2.9. In Vitro Gene Silencing Efficiency

Western blotting (WB) was used to quantitatively analyze the silencing efficiency of targeted proteins. CT26 with the complete medium were seeded into 6-well plates (5 × 10^5^ cells/well) and incubated overnight. The medium was replaced by a 2 mL fresh complete medium and 400 μL TFSPEI polyplex at the *w*/*w* ratio of 50 for 4 h. Then, each well was replaced by a 4 mL complete medium, followed by additional incubation. After 48 h, cells were collected and protein content was detected by WB. To verify whether the presence of two siRNA in the combination group would affect the expression of corresponding proteins mutually, i.e., produce synergistic or antagonistic effects, except for the target protein detection TFSPEI polyplex group, the TFSPEI-combination polyplex group was also set as control. PEI 25 kDa polyplex at *w*/*w* ratio of 2 was used as the positive control, naked siRNA was used as the negative control, and PBS was used as the blank control.

#### 2.2.10. In Vivo Anti-Tumor Treatment

An in vivo CT26 tumor-bearing mouse model was established to evaluate the anti-tumor effect of TFSPEI polyplex. Twenty-four BALB/c mice (male, 20 ± 2 g) were allocated to four groups including blank group (saline), TFSPEI-anti-PD-L1 group, TFSPEI-anti-VEGF group, and TFSPEI-combination group randomly. Then, a 0.1 mL CT26 cell suspension (5 × 10^6^/mL) was subcutaneously injected into the right subaxillary of the mice. When the tumor volume ranged from 100~150 mm^3^, mice in each group were given 0.1 mL of the corresponding solution for intra-tumor injection every 3 days. The length (L) and width (W) of the tumor and the body weight of mice were recorded. The tumor volume (V = W^2^ × L/2) was calculated synchronously with the injection time. The mice were sacrificed 3 weeks after the first injection, and the tumors were extracted and weighed, while the organs including heart, liver, spleen, lung, and kidney were extracted.

#### 2.2.11. In Vivo Anti-Tumor Effects

The blood vessels amount of tumor sections were stained by CD31 immunofluorescence and CD34 immunohistochemical to assess the tumor suppression effect. The corresponding expressions CD8 in tumor sections were analyzed by immunohistochemical staining to evaluate the regulatory effect of each treatment group on immune-related factors. For immunohistochemical staining, the tumor slices were treated with different primary antibodies according to the protocols, including CD31 antibody (Abcam, Cambridge, UK), CD34 antibody (Abcam, Cambridge, UK), and CD8 antibody (Abcam, Cambridge, UK). Three regions of each section were chosen randomly and quantitatively analyzed by Image-Pro Plus software. The expression of the target protein in tumor tissues was quantitatively evaluated by WB.

#### 2.2.12. In Vivo Cytotoxicity

The extracted organs were immersed into 4% paraformaldehyde to fix for 24 h. After fixation, the organs were stained with hematoxylin and eosin (H&E staining) to evaluate in vivo cytotoxicity.

#### 2.2.13. Statistical Analysis

The data were presented as mean values ± standard deviation (S.D.). Statistical analysis was tested by independent sample *t*-test and one-way ANOVA for multiple comparisons. Statistical analyses were performed using GraphPad Prism (San Diego, CA, USA) and significance was defined as follows: * *p* < 0.05, ** *p* < 0.01, and *** *p* < 0.001.

## 3. Results and Discussion

### 3.1. Characterization of TFSPEI

**Figure 2:**
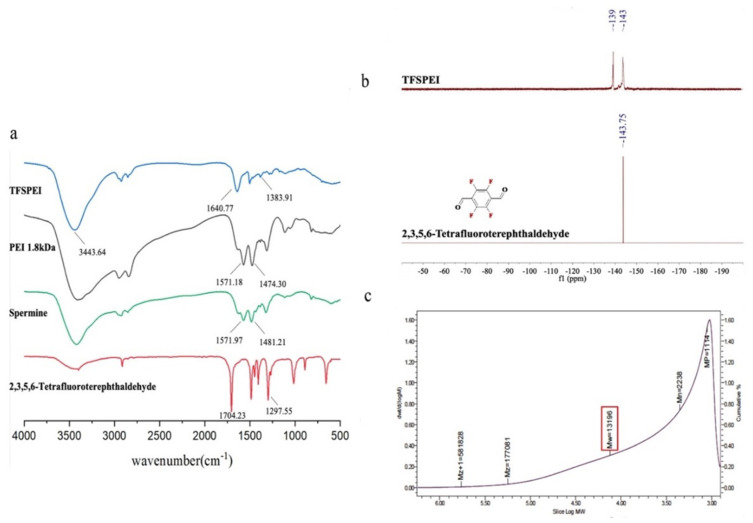
The FTIR (**a**), 19F NMR spectra (**b**), and SEC (**c**) were obtained for TFSPEI analysis.

TFSPEI was synthesized by condensing PEI 1.8 kDa and spermine with 2,3,5,6-tetrafluoroterephthaldehyde through imine linkage. First, a few spermines were reacted with 2,3,5,6-tetrafluoroterephthaldehyde to construct an oligomer short chain. Spermine contains intermediate transition link groups that can expose fluorine atoms. After that, an excess of small molecular weight PEI 1.8 kDa was added to the reaction solution, and the oligomeric further reacted with PEI 1.8 kDa using the exposed aldehyde groups at both ends, which not only links all fluorine atoms to the final product but increases the molecular weight and overall charge density. As shown in Figure 2a, the strong absorption peak of 1704 cm^−1^ in 2,3,5,6-tetrafluoroterephthaldehyde was attributed to the –C=O– bond, while there was no such absorption peak in TFSPEI, indicating that the aldehyde group had been completely reacted. Compared with PEI 1.8 kDa, TFSPEI had an obvious peak of –C=N– at 1640 cm^−1^, whose characteristic infrared absorption peak range was at 1690~1630 cm^−1^, indicating the occurrence of the dehydration condensation reaction. The absorption peak of 1383 cm^−1^ was shown in the spectrum, indicating the presence of the C-F bond.

**Figure 3:**
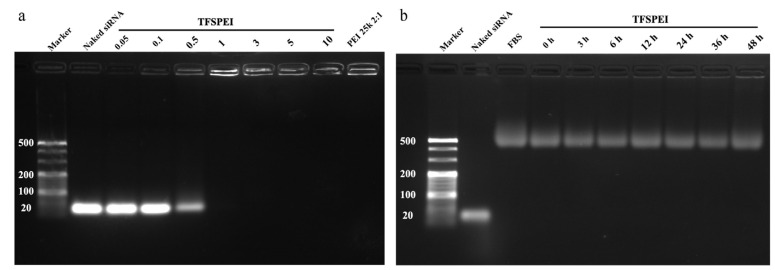
(**a**) AGE of the TFSPEI polyplex with different *w*/*w* ratios (0.05, 0.1, 0.5, 1, 3, 5, and 10) and (**b**) serum stability of the TFSPEI polyplex at the *w*/*w* ratio of 50 at different incubation times (0 h, 3 h, 6 h, 12 h, 24 h, 36 h, and 48 h).

From Figure 2b, 2,3,5,6-tetrafluoroterephthaldehyde has a unique peak at −43.75, which was produced by the fluorine atoms at the four equivalent positions in its structure. In the spectra of TFSPEI, the two chemical shifts were −139.16 and −143.58, respectively. This is due to the two aldehyde groups on the benzene ring linked to different groups, in line with the expected structure of the product. The 19F NMR spectrum was consistent with FTIR, which also proved the presence of the C-F bond, indicating the successful synthesis of TFSPEI. The weight average molecular weight (Mw) and the number average molecular weight (Mn) of TFSPEI determined by SEC were 13.196 kDa and 2.238 kDa, respectively (Figure 2c).

### 3.2. AGE and Serum Stability

The ability of carriers to condense siRNA into nanoparticles and to avoid genes being degraded before entering cells are prerequisites for gene delivery [34]. As shown in Figure 3a, when the *w*/*w* ratio was higher than 1, no electrophoresis strips of siRNA appeared, indicating that TFSPEI could successfully condense and protect siRNA at a low ratio. In addition, the stability of the complex in 10% FB was also tested. Because in a biological environment, the complex works in an environment-containing serum, large amounts of negatively charged substances may bind to the complex and destroy its structure. No siRNA strips appeared in Figure 3b, which shows that TFSPEI polyplex had high stability in FBS and could effectively protect siRNA from leakage within 48 h.

### 3.3. Zeta Potential, Particle Size, and Morphology Measurements

**Figure 4:**
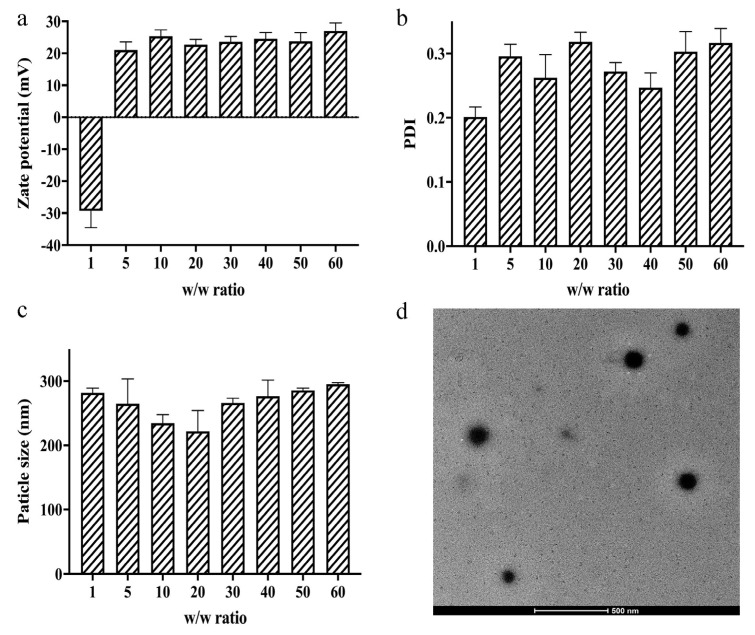
Characterization of TFSPEI polyplexes. (**a**) Zeta potential, (**b**) PDI, and (**c**) particle size of the polyplex. Data are shown as the mean ± S.D. (n = 3). (**d**) TEM images of the polyplex at the *w*/*w* ratio of 50.

Zeta potential can evaluate the stability of polyplex in solution and whether it is easy to enter cells through the cell membrane. If the overall potential of the polyplex is too high, it will be excessively electrostatically combined with the phospholipid bilayer of the cell membrane, resulting in greater cytotoxicity. As shown in Figure 4a, when the *w*/*w* ratio was greater than 5, the potential of the TFSPEI polyplex is in the range of 20–30 mV and is relatively stable. At the same time, the PDI of the polyplex uniformity was concentrated at 0.2~0.3 (Figure 4b), indicating that the uniformity was suitable with little difference under different *w*/*w* ratios. The polyplex should be successfully endocytosed into the cell to play the subsequent silencing effect, which is highly related to the particle size. The hydrodynamic diameter of TFSPEI polyplex was concentrated at 200~300 nm (Figure 4c), which was within the range that can effectively contact the cell membrane and can be easily ingested. The morphology photograph (Figure 4d) more intuitively shows that at the *w*/*w* ratio of 50, the particle size of TFSPEI polyplex showed a similar spherical shape with the range of 100~200 nm. Since DLS measures the hydrodynamic diameter of the polyplexes, the results of particle size are slightly different from dynamic particle size.

### 3.4. In Vitro Cytotoxicity

**Figure 5:**
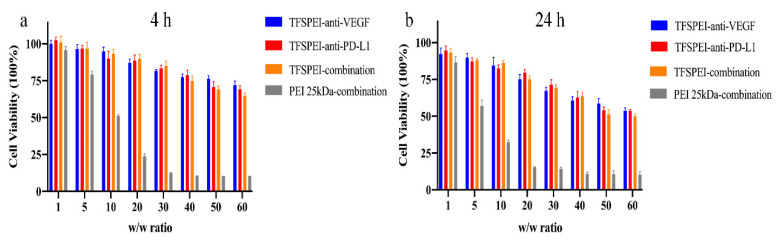
Cell viability of CT26 of four groups (TFSPEI-anti-VEGF polyplex, TFSPEI-anti-PD-L1 polyplex, TFSPEI-combination polyplex, and PEI 25 kDa-combination polyplex) at different *w*/*w* ratios (1, 5, 10, 20, 30, 40, 50, 60) incubated for (**a**) 4 h and (**b**) 24 h. Data are shown as the mean ± S.D. (n = 6).

CCK-8 was used to quantitatively determine the effect of the polyplex on cell survival rate in different periods (4 h and 24 h). As shown in Figure 5, the cytotoxicity of different polyplex groups increased with the increase of the *w*/*w* ratio. In the 4 h experiment, the cell survival rate of the TFSPEI polyplex was about 70% when *w*/*w* = 50/1. After 24 h, more than 50% of the cells in this group were still alive. Compared with TFSPEI polyplex, the cytotoxicity of the positive control PEI 25 kDa was significantly increased. In the 4-h experiment, the cell survival rate was less than 25% when *w*/*w* was greater than 20/1, and almost no cell survival was observed when *w*/*w* was greater than 30/1. This result was more obvious in the 24-h toxicity experiment. It shows that compared with PEI 25 kDa, TFSPEI has better biocompatibility and has less effect on cell survival during the process of siRNA delivery, which provides a feasible basis for subsequent application in animal experiments. In addition, the cytotoxicity of the three kinds of siRNA condensed by TFSPEI was similar. This suggests that among the siRNA selected in this study, the cytotoxicity of TFSPEI polyplex after being endocytosed may be related to the properties of the material.

### 3.5. In Vitro Endocytosis Efficiency

**Figure 6:**
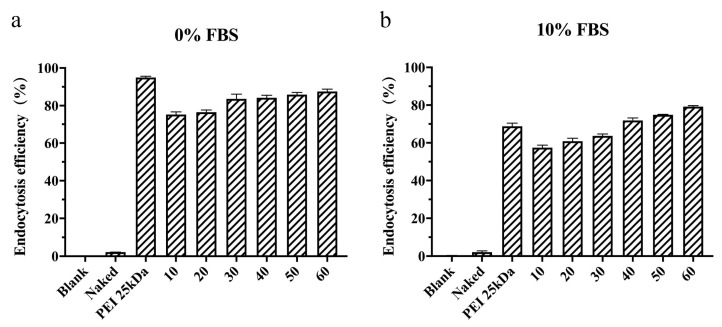
Endocytosis efficiency of TFSPEI polyplex incubated for 4 h at different *w*/*w* ratios (10, 20, 30, 40, 50, 60) with (**a**) 0% FBS and (**b**) 10% FBS. Data are shown as the mean ± S.D. (n = 3).

The ability of the polyplex to be taken up and endocytosed into the cell is the basis of their silencing function in cells [48]. As shown in Figure 6, the endocytosis efficiency of the naked siRNA group was extremely low, which was similar to that of the blank cells, indicating that it was difficult for naked siRNA to enter cells autonomously. In the serum-free environment, both PEI 25 kDa and TFSPEI polyplex showed high endocytosis efficiency, and the endocytosis efficiency of TFSPEI polyplex was positively correlated with the *w*/*w* ratio. In the environment containing 10% FBS, the endocytosis efficiency of PEI 25 kDa polyplex was significantly reduced to less than 70%, which for the TFSPEI polyplex was also reduced. However, the endocytosis efficiency of the TFSPEI polyplex was higher than that of the positive control when the *w*/*w* ratio reached 40. This result shows that compared with PEI 25 kDa, the fluorine-modified carrier has better serum stability and maintains a higher endocytosis efficiency of the system. Based on the cytotoxicity and endocytosis efficiency of TFSPEI polyplex, the *w*/*w* ratio of TFSPEI polyplex was set at 50 for subsequent cell and animal experiments. At this ratio, the endocytosis efficiency of the polyplex in the presence of serum was higher than that of the positive control PEI 25 kDa, and more than 50% of the cells were still alive in the 24 h cytotoxicity experiment.

### 3.6. Intracellular Uptake

**Figure 7:**
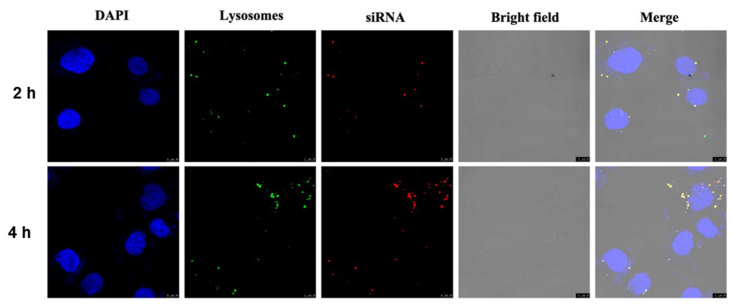
Intracellular uptake images of TFSPEI polyplex in CT26 incubated for 2 h and 4 h. Bars = 10 μm. (blue: DAPI; green: LysoTracker Green; red: Cy5-labeled siRNA).

The state of the polyplex after endocytosis was qualitatively observed under confocal laser microscopy (Figure 7). To explore the effect of endocytosis time on endocytosis efficiency, two experimental groups of 2 h and 4 h were set up. Compared with the 2 h group, the number of endosomes and lysosomes labeled with green fluorescence and the polyplex labeled with red fluorescence increased in the 4 h group, and the two superimposed in the same position showed yellow fluorescence. The results indicate that more polyplex successfully entered the acidic organelles at 4 h than at 2 h. In addition, there was no red fluorescence in the nucleus labeled with blue, which proved that the active site of siRNA was the cytoplasm and would not be delivered into the nucleus. These results suggest that the TFSPEI complex can be successfully endocytosed into cells and protect siRNA from degradation.

### 3.7. In Vitro Gene Silencing Efficiency

**Figure 8:**
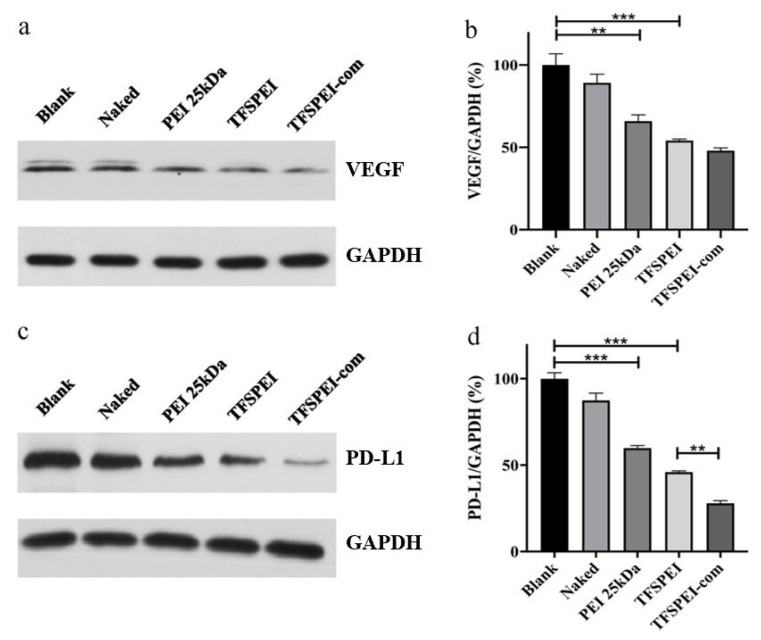
In vitro silencing efficiency of the VEGF and PD-L1 gene. The silencing efficiency of the VEGF (**a**) treated by Western blotting and (**b**) was quantitatively analyzed by ImageJ Pro. The silencing efficiency of the PD-L1 (**c**) treated by Western blotting and (**d**) was quantitatively analyzed by ImageJ Pro. Data are shown as the mean ± S.D. (n = 3, ** *p* < 0.01, *** *p* < 0.001).

As shown in Figure 8a,b, the protein content of the blank group was regarded as 100%, and the protein content of the naked group showed no significant difference, indicating that naked siRNA was difficult to enter cells autonomously to play a silencing effect. The VEGF protein content in the PEI 25 kDa group and TFSPEI group was significantly decreased, and the inhibition of protein expression in the TFSPEI group was stronger, indicating that the serum stability of TFSPEI polyplex was better in the presence of serum. In addition to the improvement of the antiserum performance of hydrophobic and hydrophilic fluorine-containing groups, the polyplex formed by the fluorine-modified carrier can also maintain a high endosomal escape ability in the serum environment. Compared with the TFSPEI group, there was no significant change in VEGF protein content in the TFSPEI-com group, indicating that there was no relevant pathway for anti-PD-L1 siRNA to synergically inhibit the expression of VEGF protein.

As shown in Figure 8c,d, the protein content of PD-L1 in the TFSPEI group was lower than that in the PEI 25 kDa group, indicating that the TFSPEI group had a stronger inhibitory effect on protein expression. Moreover, the PD-L1 protein content of TFSPEI-com was significantly lower than that of the TFSPEI group. Through quantitative analysis of WB results, it can be seen that the contents of the two groups were significantly different, confirming that anti-VEGF siRNA can synergically inhibit the expression of PD-L1 protein, providing a research basis for the combined delivery of anti-VEGF siRNA and anti-PD-L1 siRNA for gene therapy of tumors.

### 3.8. In Vivo Anti-Tumor Effects

**Figure 9:**
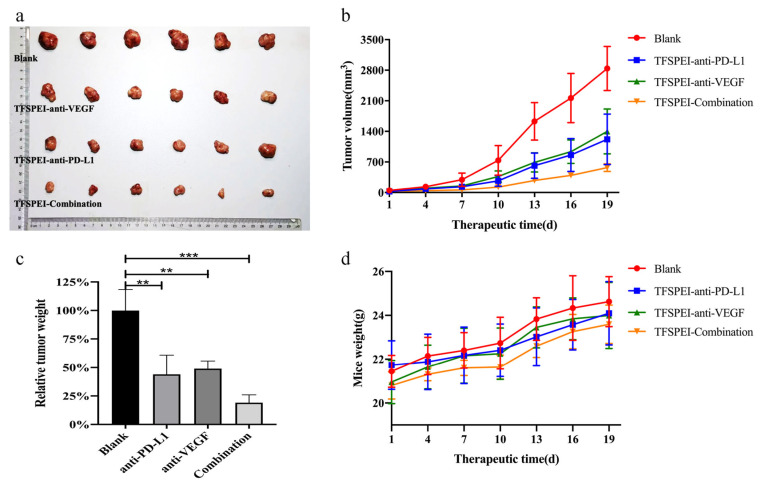
Evaluation of anti-tumor effects in vivo. (**a**) Photograph of the extracted tumors in each group on the 21st day. (**b**) The tumor volume growth curve (**c**) relative tumor weight and (**d**) weight growth curve of mice. Data are shown as the mean ± S.D. (n = 6, ** *p* < 0.01, *** *p* < 0.001).

After 3 weeks of administration, the mice were sacrificed and the tumors were extracted. The final tumor growth results in each group are shown in Figure 9a–c. With the prolongation of treatment time, the tumor volume in the blank group increased rapidly, finally growing to about 2800 mm^3^. Tumor growth was significantly slower in the treatment group, and the TFSPEI-Combination group was the slowest. The tumor volume growth trend of the TFSPEI-anti-VEGF group and the TFSPEI-anti-PD-L1 group was similar. It shows that the TFSPEI polyplex can effectively inhibit tumor growth and the therapeutic effect of simultaneously delivering anti-PD-L1 siRNA and anti-VEGF siRNA was better than that of a single siRNA. Taking the average weight of the tumor in the blank group as 100%, the tumor weight in the TFSPEI-combination group was the lowest, only about 25% of the blank group. In Figure 9d, with the increase of treatment time, the bodyweight of mice in each group increased continuously, and the overall trend was consistent, with little difference in body weight. The body weight growth curve of the mice indicated that the drug administration did not affect the growth state of the mice, and the TFSPEI polyplex used in the experiment had good biocompatibility and safety.

### 3.9. In Vivo Anti-Tumor Effect Analysis

**Figure 10:**
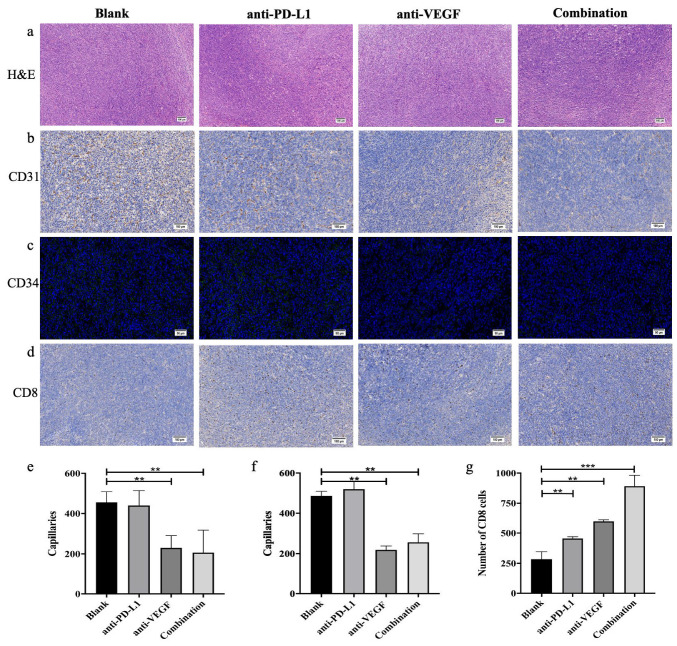
(**a**) H&E staining images of tumors. CD31 (**b**), CD34 (**c**), and CD8 (**d**) immunohistochemical staining images of tumors. Quantification of capillaries was analyzed by ImageJ Pro (**e**): CD31, (**f**): CD34). (**g**) Image-Pro Plus was used to analyze the positive rate of CD8. Data are shown as the mean ± S.D. (n = 3, ** *p* < 0.01, *** *p* < 0.001).

According to the H&E staining of tumor tissue in Figure 10a, the color and morphology of tumor tissues in each group were consistent without significant difference, and the cell growth was dense and vigorous. The results indicate that the tumor types in each group are consistent and no abnormal tumor tissue exists. CD31 and CD34, as the markers of endothelial tissue differentiation, can be used to evaluate tumor angiogenesis [49,50]. As shown in Figure 10b,c, the nuclei were marked blue, the CD31-positive areas were marked dark brown, and CD34-positive areas were marked green. There were many positive signals in the blank group and the TFSPEI-anti-PD-L1 group, and the overall distribution was uniform and dense, indicating that there were more new blood vessels and vigorous tumor growth. The positive signal density was significantly reduced in the TFSPEI-anti-VEGF group and the TFSPEI-combination group, and the difference between the two groups was not significant. These results show that anti-VEGF siRNA delivered by TFSPEI can effectively inhibit tumor angiogenesis, and there is no relevant pathway for anti-PD-L1 siRNA to synergically inhibit the expression of VEGF protein. Image-Pro Plus was used for semi-quantitative analysis of the number of blood vessels, and the average number of new blood vessels was consistent with the above observations (Figure 10e,f).

CD8 is an antigen that can characterize the specific killing activity of T cells against target cells [51]. The more CD8-positive cells, the stronger the killing and invasion effect of T cells against the tumor. As shown in Figure 10d, the nuclei were marked blue, and CD8-positive areas were marked dark brown. The blank group had the least positive signals, with loosely distributed and small areas. The positive signals in the TFSPEI-anti-VEGF group and the TFSPEI-anti-PD-L1 group were significantly higher than those of the blank group, indicating that the specific killing activity of T cells to target cells was restored to a certain extent. The TFSPEI-combination group had the highest positive signal density, indicating that silencing the expression of PD-L1 and VEGF on the surface of tumor cells with dual signal pathways can achieve bidirectional targeted therapy of tumors and synergistically enhance the anti-tumor effect. Image-Pro Plus was used for semi-quantitative analysis of the number of CD8-positive cells, the result was consistent with the above observations (Figure 10g).

**Figure 11:**
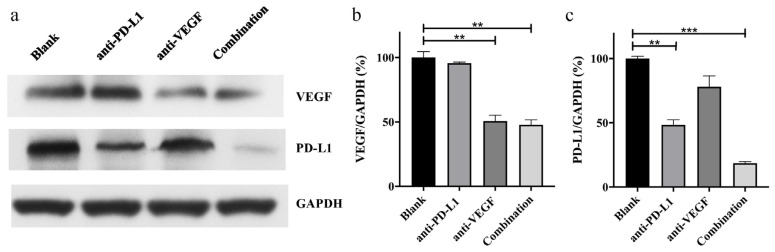
In vivo gene silencing efficiency of VEGF and PD-L1. (**a**) Western blotting was used to indicate the expression of VEGF and PD-L1. The expression of VEGF (**b**) and PD-L1 (**c**) was quantitatively analyzed by Image J. Data are shown as the mean ± S.D. (n = 4, ** *p* < 0.01, *** *p* < 0.001).

The inhibition effect of target protein content, the final expression form of the delivery system at the cell level, was measured by the WB experiment, to directly evaluate the ability of TFSPEI polyplex to deliver siRNA to inhibit the expression of related proteins. As shown in Figure 11a, at the VEGF level, the protein content of the TFSPEI-anti-PD-L1 group was similar to that of the blank group, while the protein content of the TFSPEI-anti-VEGF group was significantly reduced. This result suggested that anti-VEGF siRNA delivered by TFSPEI could effectively inhibit the expression of VEGF protein. Compared with the TFSPEI-anti-VEGF group, the amount of protein in the TFSPEI-combination group did not significantly change, indicating that there was no relevant pathway for anti-PD-L1 siRNA to synergically inhibit the expression of VEGF. At the PD-L1 level, the protein content of the TFSPEI-anti-PD-L1 group decreased, while the TFSPEI-combination polyplex group further decreased, indicating that the simultaneous delivery of anti-VEGF siRNA and anti-PD-L1 siRNA through TFSPEI can play a synergistic role, effectively restore the lethality of T cells and inhibit tumor growth. Taking the result of blank group protein content as 100%, the specific value obtained by semi-quantitative protein analysis with Image J was consistent with the above observations (Figure 11b,c).

### 3.10. In Vivo Cytotoxicity

**Figure 12:**
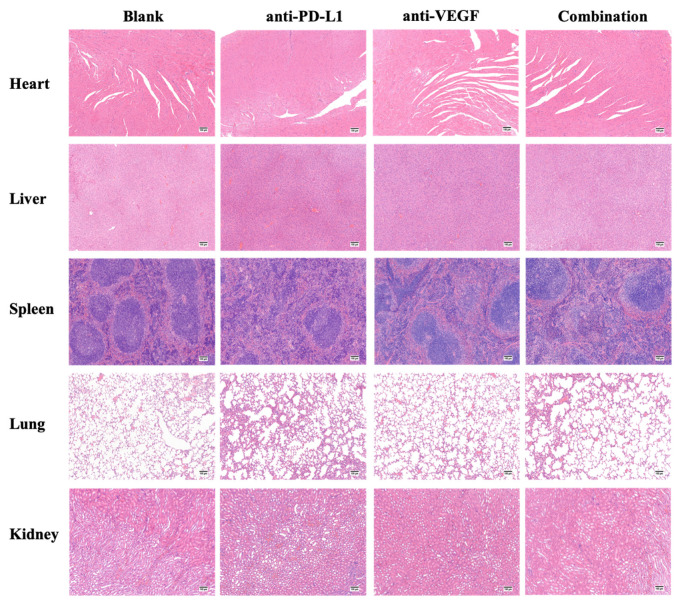
Evaluation of in vivo toxicity to main organs. H&E staining images of the main organs. Bars = 100 μm.

Histopathological biopsy of main organs is the most visual way to observe tissue morphology and judge the pathological changes of organs. As shown in Figure 12, the color and tissue morphology of each group were normal and similar, and no abnormal areas such as tissue necrosis appeared, indicating that the TFSPEI polyplex used in each treatment group had good biocompatibility and safety, and would not cause obvious in vivo toxicity to organisms.

## 4. Conclusions

In this study, fluoride TFSPEI was successfully constructed by forming an imine linkage between PEI 1.8 kDa, spermine, and the aldehyde group. The carrier can condense siRNA at a low *w*/*w* ratio and protect them from degradation, with ideal uniformity and serum stability. Compared with PEI 25 kDa, TFSPEI has lower toxicity, better biocompatibility, and higher endocytosis and silencing efficiency in the serum environment. In the study of solid tumor models, the delivery of anti-VEGF siRNA and anti-PD-L1 siRNA can inhibit the expression of corresponding proteins, restore the anti-tumor function of T cells, and inhibit the growth of new blood vessels. The therapeutic effect of simultaneously delivering anti-PD-L1 siRNA and anti-VEGF siRNA was better than that of a single siRNA. In addition to the inherent anti-angiogenesis mechanism, the inhibition of VEGF targets can indirectly activate the immune response to promote the tumor-killing and invasion effects of T cells, which can produce synergistic anti-tumor effects when combined with immunotherapy. Consequently, we believe that a more practical approach will be the combined delivery of anti-PD-L1 and anti-VEGF siRNA through TFSPEI polyplex because it has efficient gene delivery and can realize bidirectional targeted therapy of tumors.

## Data Availability

Not applicable.
